# Young Japanese Underweight Women with “Cinderella Weight” Are Prone to Malnutrition, including Vitamin Deficiencies

**DOI:** 10.3390/nu15092216

**Published:** 2023-05-07

**Authors:** Katsumi Iizuka, Hiroko Sato, Kazuko Kobae, Kotone Yanagi, Yoshiko Yamada, Chihiro Ushiroda, Konomi Hirano, Satomi Ichimaru, Yusuke Seino, Akemi Ito, Atsushi Suzuki, Eiichi Saitoh, Hiroyuki Naruse

**Affiliations:** 1 Department of Clinical Nutrition, Fujita Health University, Toyoake 470-1192, Japan; chihiro.ushiroda@fujita-hu.ac.jp; 2 Food and Nutrition Service Department, Fujita Health University Hospital, Toyoake 470-1192, Japan; konomi@fujita-hu.ac.jp (K.H.); stm.ichimaru@gmail.com (S.I.); akemi305@fujita-hu.ac.jp (A.I.); 3 Health Management Center, Fujita Health University, Toyoake 470-1192, Japan; hsato@fujita-hu.ac.jp (H.S.); kobae@fujita-hu.ac.jp (K.K.); yanagi-k@fujita-hu.ac.jp (K.Y.); yoshiko.yamada@fujita-hu.ac.jp (Y.Y.); hnaruse@fujita-hu.ac.jp (H.N.); 4 Department of Endocrinology, Diabetes, Metabolism, Fujita Health University, Toyoake 470-1192, Japan; seinoy@fujita-hu.ac.jp (Y.S.); aslapin@fujita-hu.ac.jp (A.S.); 5 Department of Rehabilitation Medicine I, School of Medicine, Fujita Health University, Toyoake 470-1192, Japan; esaitoh1@me.com; 6 Department of Medical Laboratory Science, Fujita Health University Graduate School of Health Sciences, Toyoake 470-1192, Aichi, Japan

**Keywords:** undernutrition, dietary diversity score, 25-OH vitamin D

## Abstract

Undernutrition among young women at “Cinderella weight” is socially important in Japan. To determine the nutritional status of Cinderella-weight women, we conducted an exploratory cross-sectional study on the health examination results of employees aged 20 to 39 (n = 1457 and 643 for women and men, respectively). The percentage of underweight women was found to be much higher than that of men (16.8% vs. 4.5%, respectively). In underweight women (n = 245), handgrip strength (22.82 ± 5.55 vs. 25.73 ± 5.81 kg, *p* < 0.001), cholesterol level (177.8 ± 25.2 vs. 194.7 ± 31.2 mg/dL, *p* < 0.05), and lymphocyte count (1883 ± 503 vs. 2148 ± 765/μL, *p* < 0.001) were significantly lower than in overweight women (n = 116). Then, the BMI < 17.5 group (n = 44) was referred to the outpatient nutrition evaluation clinic. Lower prealbumin, cholesterol, and lymphocyte levels were also observed in 34%, 59%, and 32% of the patients, respectively. Regarding dietary characteristics, 32% of the underweight women in this study skipped breakfast, and 50% had low dietary diversity scores. Lower total energy intake, carbohydrate and fiber intake, and Ca and Fe intake were also observed in 90% of the patients. Deficiencies in vitamin B_1_, B_12_, D, and folate were diagnosed in 4.6%, 25%, 14%, and 98% of the patients, respectively. Thus, young underweight women may be prone to malnutrition.

## 1. Introduction

The definition of malnutrition includes both undernourished and overnourished status [1]. In Japan, overweight and obesity are well-known cardiovascular risk factors and have received attention [2]. In contrast, being underweight is often overlooked in physical checkups. Undernutrition is often seen in poverty and social isolation and is associated with severe diseases [1]. Moreover, undernutrition causes muscle function decline, decreased cardiorespiratory function, and decreased digestion [1]. Several nutrition screening tools have been developed, and now, Global Leadership Initiative on Malnutrition (GLIM) criteria are widely used to diagnose malnutrition [3].

The word “Cinderella weight” originated in Japan and implies a BMI of 18 [4]. The percentage of young underweight women in Japan was 21.5% in 2013, while that in the USA was 2% [5,6]. The problem with the “Cinderella weight” condition is that, while appearing harmless, it is potentially complicated by the risks associated with undernutrition [1,7,8,9,10,11]. For example, the risks associated with undernutrition include menstrual abnormalities, infertility, and osteoporosis. Furthermore, children born to underweight individuals have low birth weight, which increases the risk of developing diabetes and cardiovascular disease in the future [1]. For example, a maternal diet with many UPFs, saturated fats, and total sugars (especially those added or hidden in packaged carbonated beverages) can adversely affect a child’s cognitive development [12]. In addition, folic acid deficiency is associated with the risk of fetal spina bifida, and low vitamin D levels are associated with an increased risk of complications in pregnant women and their babies [1,10,11,12,13]. Low folate may increase cardiovascular risk because it increases homocysteine levels [14].

Additionally, weight loss may be due to hyperthyroidism and other conditions. Therefore, although a differential diagnosis of malnutrition and nutritional therapy for underweight patients is necessary, a lack of symptoms may prevent patients from seeking medical attention. Thus, the full picture of the health risks that young individuals with malnutrition have remains unclear.

The health risks associated with loss of dietary diversity and breakfast deprivation have long been reported [15,16,17,18,19]. For example, dietary diversity is substantially related to age-specific height Z score (HAZ), and there is an association between dietary diversity and nutritional status in children independent of socioeconomic factors, indicating that dietary diversity may reflect dietary quality [15]. Higher dietary diversity scores were significantly associated with a lower risk of MetS and abdominal obesity [16]. In addition, the dietary diversity score (DDS) is positively correlated with indicators of micronutrient sufficiency, with scores of 6 to 8 indicating the lowest risk of micronutrient deficiencies in different groups of children [17]. It has also been reported that breakfast skipping is associated with obesity [18]. Breakfast consumption was also associated with higher dietary quality index scores and lower body mass index and waist circumference values among young women in Isfahan [19]. Thus, the dietary diversity score and the absence of breakfast indicate food quality.

In this study, we hypothesized that underweight, as well as obesity, is an important health risk. However, since we do not know the actual situation regarding the nutritional status of Cinderella weight, we conducted the following study. First, we examined the proportion and characteristics of underweight women who attended workplace health examinations. Next, body composition, muscle strength, response to the food frequency questionnaire, protein synthesis, and plasma vitamin levels were investigated in patients with a BMI of less than 17.5 who visited the outpatient nutrition evaluation clinic. This study will clarify the health status of underweight young women whose actual conditions have not been fully elucidated.

## 2. Materials and Methods

### 2.1. Study Design and Participants

This study was a retrospective cross-sectional study. Men (n = 643) and women (n = 1457) aged 20–39 years who received workplace health examinations (from August 2022 to September 2022) were included. Next, 56 female patients visited our department for a second screening due to low body weight (BMI < 17.5) from November 2022 to February 2023. A more in-depth study was conducted on 44 female individuals aged 20–39 and 12 female individuals aged 40–65 who visited our department for secondary screening. The reason the 20–39 age group was chosen as the fertile age group is that many people in this generation are unmarried and live alone, and their social background is clearly different from that of the 40+ age group. This is also the age when vitamins such as folic acid are especially needed for pregnancy.

A total of 91% and 75% of patients aged 20–39 years old and 40–65 years old, respectively, qualified as undernourished by GLIM criteria [3] due to low BMI and low dietary intake. Pregnant women and patients being seen in psychiatry for anorexia nervosa were not included because they will be seen by their primary care physician. The presence or absence of fever, intentional weight loss, and appetite were investigated by interview, but there were no obvious abnormalities. Most cases were not noted on physical examination except for low body weight. Thyroid function was also examined in all cases seen. Menstrual abnormalities were not found in most cases. Since the patients ate only enough to maintain their current weight, it is unlikely that their low weight was due to some underlying disease.

### 2.2. Data Collection and Food Frequency Questionnaires

For males aged 20–39 years who underwent health checkups, anonymized BMI, gender, and age data were provided by the Health Management Department. Anonymized data on BMI, sex, age, grip strength, blood pressure, HbA1c, cholesterol, and lymphocyte count were provided by the outpatient nutritional evaluation for women aged 20–39 who underwent health checkups.

For the 56 female patients who visited our department for secondary screening, age, sex, BMI, BMI at age 20, height, weight, blood pressure, grip strength, body fat percentage, SMI, and blood data (HbA1c, Hb (hemoglobin), TSH (thyroid stimulating hormone), free thyroxin (FT4), C-reactive protein (CRP), prealbumin, albumin, cholesterol, lymphocyte count, vitamin B1, vitamin B_12_, folic acid, and 25-hydroxyvitamin D (25(OH)D)) were obtained from medical records. Body weight, body fat percentage, and skeletal muscle mass index (SMI) were calculated with height and skeletal muscle mass using InBody Dial H20N (Inbody Japan Inc., Tokyo, Japan). A food frequency questionnaire (FFQ) was also administered using FFQg version 6 software (Kenpakusha, Tokyo, Japan) at the first outpatient visit to calculate macronutrient and micronutrient intake [20,21,22], the presence of breakfast deprivation, and the food diversity score [23,24]. This FFQ is developed for Japanese people, and questions are asked in relation to Japanese food (Japanese and Western). FFQg is based on 29 food groups and 10 types of cooking and is for estimating the energy and nutrient intakes of an individual subject during the past 1–2 months. The diversity of food intake was determined using the dietary diversity score (DDS). The respondents were asked about approximately 10 food groups (meat, seafood, eggs, milk, soy products, green and yellow vegetables, seaweed, fruits, potatoes, and fats and oils) using a 4-point scale, where the responses include “Eat every day,” “Eat once every two days,” “Eat 1–2 times per week,” and “ Eat rarely” (the total score (out of 10) is used to calculate the diversity score of food intake) [23,24]. Only the response of "Eat every day" is counted as 1 point, and all others are counted as 0 points. A total score of 7–10 points is high, 4–6 points is medium, and 0–3 points is low.

In accordance with a previous paper, the CONUT score, a screening tool for detecting inadequate nutrition based on low albumin, cholesterol, and lymphocytes, was calculated using Alb, cholesterol, and lymphocytes [25].

### 2.3. Statistical Analysis

Categorical variables are represented as numbers (percentages), and parametric variables are defined as the means ± standard deviations. Body composition (undernutrition, normal, and overnutrition) was compared between men and women using the χ-squared test and Student’s *t*-test.

Regarding the background of young women, the χ-squared test was performed for categorical variables (HbA1c ≥ 5.6%, HbA1c ≥ 6.0%, cholesterol < 180 mg/dL, lymphocyte count < 1600/μL, Hb < 12 g/dL, prealbumin < 22 mg/dL, Alb < 4 g/dL, CONUT score, breakfast skipping, DDS, total energy < 2050 kcal, protein < 50 g, fat < 46 g, carbohydrate < 256 g, dietary fiber < 18.9 g, *n*-3 PUFA < 1.6 g/day, *n*-6 PUFA <8 g/day, vitamin B_1_ intake (<1.1 mg), plasma vitamin B1 deficiency (<24 ng/mL), vitamin B_12_ intake (<2.0 μg), vitamin B_12_ deficiency (<200 pg/mL), folate intake (<240 μg), plasma folate deficiency (<4 ng/mL), vitamin D intake (<8.5 μg), and plasma 25(OH)D deficiency < 20 ng/mL) [26]. One-way ANOVA and Tukey’s test were performed for parametric variables (age, BMI, BMI at 20 years of age (y.o.a.), handgrip strength, skeletal muscle index, calf circumference, body fat %, HbA1c (%), cholesterol, lymphocyte count, Hemoglobin (Hb), TSH, FT_4_, CRP, Alb, prealbumin, total energy expenditure (TEE), and total energy intake (TEI)).

## 3. Results

### 3.1. Underweight Is Much More Frequently Seen in Young Women Than in Young Men

First, we compared the body composition of young women (20–39 years old, n = 1457) with that of underweight young men (20–39 years old, n = 643). The proportion of underweight young women (n = 245, 17%) was much higher than that of underweight young men (n = 29, 4.5%). In particular, the proportion of very underweight women (BMI < 17.5) was 5.9% (n = 86), while that of very underweight men was 1.4% (n = 9). In contrast, the proportion of young overweight women (n = 116, 8.4%) was much lower than that of young overweight men (n = 132, 20.5%) (Table 1). Thus, lower body weight in young female subjects is an important health problem compared with that in young male subjects.

We then classified the young women into underweight, normal, and overweight groups and examined indices reflecting nutritional status, such as age, BMI, grip strength, blood pressure, HbA1c, cholesterol, and lymphocytes. Age and BMI were significantly higher in the normal and overweight groups than in the underweight groups. On the other hand, grip strength was significantly lower in the underweight group than in the normal and overweight groups. Blood pressure and HbA1c were significantly higher only in overweight subjects, with no significant difference between normal and underweight subjects. Cholesterol and lymphocytes are markers of nutritional status. Both cholesterol and lymphocytes were significantly lower in the underweight group, and the percentages corresponding to cholesterol <180 mg/dL and lymphocytes <1600/μL were lowest in the underweight group (Table 2).

Thus, in the underweight group, there was a decrease not only in BMI but also in markers reflecting nutrition, such as grip strength, cholesterol, and lymphocytes. On the other hand, HbA1c values did not differ from those in the normal weight group. Thus, underweight subjects tend to be characterized as malnourished.

### 3.2. Clinical Parameters of Patients Admitted to the Outpatient Nutritional Evaluation

Next, among 88 and 24 subjects who were identified as underweight (BMI < 17.5) from the health examination results of employees aged 20 to 39 at Fujita Health University Hospital, the 56 patients with BMI < 17.5 referred to our department included 44 young women aged 20–39 years and 12 women aged 40 years and older, with BMIs of 16.96 ± 0.67 and 17.27 ± 0.71, respectively (Table 3). The results of t-tests showed no difference between the two groups. BMIs at 20 y.o.a. were similar between both groups (Table 3). Grip strength was also low in both groups, less than 24, and tended to be lower in younger women.

Leg circumference decreased to approximately 31 cm in both groups, and body fat percentage was normal and low in both groups. There was no difference in these indices between the two groups.

Hb did not differ between the two groups, but the proportion of those diagnosed with anemia was higher in the younger group (16% and 8.3%), although not significantly so; HbA1c was normally high in both groups (5.5%), and the proportion of those with HbA1c of 5.6% or higher in the younger and older group was 36% and 50%, respectively. Thyroid function was normal, and CRP was negative. However, 4 patients (2 patients in each group aged 20–39 and above 40 years old) were diagnosed with TRAb-positive Graves’ disease. Prealbumin is one of the indicators of protein synthesis. Both groups, younger and older, had normal low levels, which were decreased by 34% and 25%, respectively. On the other hand, albumin was normal, averaging 4.5, the percentages of patients with low albumin were 4.5% and 8.3%, respectively, and the portion was higher in patients over 40. On the other hand, low cholesterol levels were seen in both younger and older groups, 59% and 5, respectively. The CONUT score, a screening tool for detecting inadequate nutrition based on low albumin, cholesterol, and lymphocytes, was more common in patients aged 40 and older, with 25% and 58% of the younger and older groups with mildly high CONUT, respectively.

### 3.3. The Analysis of Food Frequency Questionnaires in Young Underweight Subjects Admitted for Outpatient Nutritional Evaluation

Next, we conducted a food intake frequency survey to investigate problems in younger and middle-aged underweight patients. Breakfast absenteeism was more common among younger patients (32% in the 20–39 age group and 17% in the 40+ age group, *p* = 0.3) (Table 4). Next, the DDS was examined, and half of both groups were rated as having low food diversity (Table 4).

When we examined the DDS for all ages of breakfast skippers and breakfast nonskippers, we found that the scores were 2.44 ± 1.87 and 4.18 ± 0.83, respectively, which were significantly higher for breakfast nonskippers (*p* = 0.006). Similarly, for those under 40, the DDS of breakfast nonskippers (4.00 ± 2.06) was considerably higher than that of breakfast skippers (2.36 ± 1.83, *p* = 0.015 vs. nonskippers). Thus, breakfast skipping was associated with lower food diversity, and breakfast skipping and lower food diversity were characteristic of underweight women.

Energy intake was approximately 1600 calories, roughly the same as the total energy consumption calculated from the Harris–Benedict equation (Table 4). Next, the intake of the three macronutrients was examined. Protein intake was lower in the 20–39 age group (32%) and in the 40+ age group (17%). Carbohydrate intake was also lower in the younger group (89%) and middle-aged group (58%). In contrast, fat intake was normal in both groups. Dietary fiber intake was lower in the 20–39 age group (95%) and in the 40+ age group (83%). Surprisingly, cholesterol intake was as high as 270–280 mg. *n*-3 PUFA and *n*-6 PUFA were lower in the 20–39 age group (34 and 30%) and the 40+ age group (25 and 25%), respectively. Fe and calcium intake were low in most of the 20–39 age group and in the 40+ age group (Table 4).

### 3.4. Vitamin Intake and Plasma Vitamin Levels in Young Underweight Female Subjects

Furthermore, we conducted FFQ to investigate the incidence of vitamin deficiency and vitamin intake (Table 5).

The intake of vitamin B_1_ was 0.89 ± 0.28 for those aged 20–39 and 0.92 ± 0.25 for those aged 40 and older, with more than 80% of the participants eating less than the recommended amount. Consistent with these results, 4.5% and 25% of the individuals, respectively, were noted to have low blood vitamin B_1_ levels (<24). The intake of vitamin B_12_ was 4.22 ± 2.92 for those aged 20–39 and 5.96 ± 2.53 for those aged 40 and older. Vitamin B_12_ intake tended to be lower in the 20–39 age group. However, blood data indicated vitamin B_12_ deficiency in 25% of the 20–39- and 40-plus-year-olds. Regarding folic acid, 80% of those aged 20–39 and 33% of those aged 40 and older did not consume the recommended amount. Consistent with this result, 14% of the 20–39-year-olds had decreased blood folate levels. Finally, vitamin D intake was low in both groups, with values of 4.12 ± 3.14 and 5.72 ± 2.65 in the 20–39 and 40+ age groups, respectively, and the percentages of those not consuming the recommended intake were 95% and 83%, respectively. Those diagnosed with low blood 25(OH)D, i.e., vitamin D deficiency, were significantly more frequently seen in the 20–39 age group (98%) and the 40+ age group (83%). Thus, young underweight subjects were frequently prone to vitamin deficiency due to their lower intake of vitamins.

## 4. Discussion

We investigated nutritional conditions such as body composition, blood parameters, and food frequency in young female subjects admitted to the checkup examination. Compared with young male subjects, the proportion of underweight individuals in young female subjects was significantly higher. Young underweight subjects showed malnutrition compared with young subjects in overweight groups. Individuals in the young underweight female group showed anemia (16%), higher HbA1c levels (36%), lower prealbumin levels (34%), lower cholesterol (59%), lower lymphocytes (32%), and a mildly high CONUT score (25%). Along with lower food intake, breakfast skipping, lower food diversity, lower total energy intake, lower protein and carbohydrate intake, lower dietary fiber intake, lower *n*-3 and *n*-6 PUFA intake, and lower Zn and calcium intake were observed. Finally, probably due to lower vitamin intake, young underweight subjects showed vitamin deficiencies such as vitamin VB_1_, B_12_, folate, and D deficiencies. Thus, these results suggested that young underweight female subjects have some nutritional problems.

Following the GLIM criteria, almost all underweight patients (BMI < 17.5, reduced dietary intake) were diagnosed with malnutrition. Underweight was due to starvation but also partly due to increased energy expenditure associated with hyperthyroidism. Aiming for a “Cinderella” weight harmfully increased the risk of irregular menstruation, infertility, cardiovascular disease, glucose intolerance, and osteoporosis. Some researchers have reported that underweight predisposes people to developing T2DM [9]. In underweight men and women, the multivariate-adjusted hazard ratios (HRs) and 95% confidence intervals (CIs) for the incidence of diabetes were 1.87 (1.04–3.36) and 1.93 (1.19–3.13), respectively [27]. Moreover, the prevalence of IGT was higher in underweight women than in normal-weight women (13.3% vs. 1.8%). Consistent with these findings, OGTT data showed that AUC glucose during OGTT was almost similar, and HbA1c levels in underweight young women were lower than those in normal-weight women. Our data showed that HbA1c levels were comparable between the underweight and normal-weight groups. These results suggested that lower body weight itself did not accelerate or ameliorate glucose intolerance, at least in young women.

Handgrip strength, total cholesterol, lymphocytes, and prealbumin are well-known markers of malnutrition. Some researchers reported that BMI was positively correlated with handgrip strength, which is consistent with our data [28,29]. Cholesterol levels were also positively associated with BMI in the case of a BMI under 25 [30]. Lymphocytes also reflect the nutritional condition [30,31]. Levels of lymphocytes have been shown to vary with the degree of malnutrition [31]. These results are consistent with our data showing that handgrip strength, cholesterol, and TLC are lowest in underweight groups of individuals who undergo a physical examination. Moreover, prealbumin is known as one of the nutritional markers and is an indicator of protein synthesis, which is reported to be decreased when total energy is low [32,33]. Prealbumin levels are lower in those with malnutrition status, and a low-energy high-protein diet also decreases prealbumin levels [33]. Indeed, in young underweight subjects admitted to our department, lower handgrip strength, lower cholesterol, and lower lymphocytes were accompanied by lower prealbumin levels. Thus, these results suggested that a lower BMI implies malnutrition in young women as well.

In contrast to prealbumin levels, plasma albumin levels were unaffected. A meta-analysis of 63 studies on the effects of starvation on serum albumin levels reported that the levels remained normal until the patients reached extreme states of starvation (BMI < 12 or length > 6 weeks of starvation) when malnutrition was already physically evident [31]. As the BMI of our subjects was as high as 16.96 ± 0.67, our results are consistent with the meta-analysis data. These results suggested that albumin cannot be a reliable marker for diagnosing protein-calorie malnutrition. Prealbumin is a rapid turnover protein and is used as a marker reflecting nutritional conditions. Feeding very low-calorie diets to overweight patients caused a decrease in prealbumin but not albumin levels. These results suggested that prealbumin, rather than albumin, is a good marker for nutritional conditions. Therefore, considering that the CONUT score is calculated with albumin, lymphocytes, and cholesterol, only lymphocytes and total cholesterol may affect the CONUT score in underweight patients.

FFQ data revealed that breakfast skipping and lower food diversity were often seen, especially in young underweight women. Breakfast skipping is reported to be associated with body weight gain [34,35,36]. Participants assigned to breakfast had a higher total daily energy intake than those assigned to skip breakfast [35]. Inconsistently, our data showed that breakfast skipping did not promote overeating (1708 ± 372 and 1469 ± 405 kcal in breakfast nonskippers and skippers, respectively, *p* = 0.08). Therefore, our results indicated that breakfast skipping may cause a decrease in total energy intake in “underweight” subjects. Moreover, many of the young underweight women in this study live alone and cook for themselves. Young women are characterized by an unbalanced diet, preferring meat dishes to fish and consuming fewer green and yellow vegetables. Some do not eat breakfast, eat only pasta or rice balls with few ingredients from convenience stores for lunch, and go to bed without eating at night. This leads to a bias in the types of foods consumed and a loss of diversity. In addition, vegetable intake is low, resulting in less fiber and fewer fish dishes. Once married, these young women are more concerned about the diet of their children and husbands, and their vegetable intake and protein intake, including fish, increase slightly. Thus, protein intake, carbohydrate intake, vitamin (B_1_, B_12_, folate, and D) intake, and mineral (Ca and Fe) intake were seen to be lower in young underweight women [35,36,37,38]. Thus, breakfast skipping and lower DDS are characteristic of the eating habits in young underweight women.

Folate intake in young underweight women is lower than that in middle-aged underweight women. Consistently, the incidence of folate deficiency was much higher in young underweight women. Decreased folate intake in young women is a social problem in Japan. Maternal folic acid deficiency has been reported to be involved in the development of fetal spina bifida and neural tube defects [10,37]. Some researchers reported that dietary folate intake and plasma concentration were highest in pregnant women (356 µg/day and 11.9 ng/mL) and lowest in student nurses (217 µg/day and 6.8 ng/mL) [37]. In our data of young underweight patients, folate intake and plasma folate levels were 204 ± 78 and 7.91 ± 5.05 ng/mL, respectively, and the standard deviation was very broad. These results suggest that it is important to educate those who do not eat vegetables rather than provide a uniform recommendation for folic acid intake [10,38,39].

Vitamin D is important to regulate and maintain bone and muscle functions through calcium and phosphate metabolism and decrease the risk of bone fracture, falls, atherosclerosis, and all-cause mortality. Vitamin D deficiency is often asymptomatic; however, vitamin D deficiency sometimes causes symptoms including bone pain, muscle weakness and cramps, fatigue, and depression. Moreover, vitamin D deficiency causes accelerated bone demineralization, such as osteomalacia and osteoporosis. Approximately 1 billion people worldwide have vitamin D deficiency, while 50% of the population has vitamin D insufficiency [40]. Approximately 35% of adults in the United States have vitamin D deficiency. In Japan, the frequencies of vitamin D deficiency in young women were 90.5%, 62.5%, 81.5%, and 91.3 in spring, summer, fall, and winter, respectively [41]. As our patients were admitted to our department from December 2022 to February 2023, these data are not contradictory to our data that 98% of young underweight patients are diagnosed with vitamin D deficiency. Moreover, serum 25(OH)D levels (10.78 ± 4.18 ng/mL) and vitamin D intake (4.12 ± 3.14) in winter were much lower than the previously reported data in winter (serum 25(OH)D levels (12 ± 5.5 ng/mL) and vitamin D intake (9.6 ± 5.1 μg/day)) [38]. Low vitamin D status is associated with decreased bone mineral density in lactating women with low BMI [42]. Moreover, as vitamin D is also stored in adipose tissues, vitamin D is also inversely associated with fat mass [43,44]. Therefore, the enlarged adipose mass in obese individuals serves as a reservoir for vitamin D, and atrophied adipose tissue has a reduced capacity to store vitamin D. These results suggest that vitamin D deficiency in young underweight women is more common and severe than in normal-weight subjects. Although the factors increasing the risk of vitamin D deficiency are age, dark-colored skin, and mobility, the risk of lower vitamin D in young women appears to be due to lower intake and less time exposed to sunlight [45]. Thus, strategies, including dietary guidance for vitamin D deficiency, especially for young thin women, are urgently needed to assure future health.

Lower intakes of other vitamins (Β_1_ and B_12_) were also observed. In particular, vitamin B1 intake was not up to standard for some individuals. No one complained of obvious neurological disorders or had macrocytic anemia, but there was a potential decrease in intake. Although vitamin B_1_ levels below 24 were observed in 4.6% and 25%, lower vitamin B1 levels (<27 ng/mL) were observed in 15 (34%) and 6 (50%) young and middle-aged underweight women, respectively. These results suggested that plasma vitamin B_1_ levels in young underweight women were relatively lower. Considering that vitamin B_1_ deficiency can cause refeeding syndrome, it appeared that supplementation with water-soluble vitamins would be necessary if the underweight patients were to undergo surgery or other invasive procedures or if they were unable to eat for 10 days.

The advantage of this study is that it reveals the reality of undernutrition in subjects who are easily not evaluated on account of their low body weight alone. Conversely, the limitations of this study are, first, that it is an observational study and cannot prove a causal relationship. Second, the number of subjects was small, and some of the data cannot be compared with those of healthy subjects. Low nutrition among young women is a problem that affects not only the women themselves but also their children; it is a social issue in Japan, involving the declining birthrate. In the future, it will be necessary to conduct a large-scale study that includes all of Japan. If so, it would need to be conducted for all young women, and comparisons with normal-weight and overweight groups would be necessary. In addition, if body composition analysis including DXA were conducted, bone mass would also be determined. Moreover, the accuracy of TEE and TEI is considered to increase if they are measured in combination with the dietary interview method and activity measurement using an activity meter in the future.

In conclusion, a higher proportion of younger women than men are underweight. Furthermore, the risk of glucose intolerance remains the same in underweight individuals, although the index reflecting nutritional status is lower than that in normal-weight individuals. Dietary intake was only in amounts necessary to maintain the current body weight, and inadequate intake of protein, carbohydrates, dietary fiber, and vitamins Β_1_ and D was observed. Vitamin deficiencies were observed, especially of folic acid and vitamin D, which have traditionally been noted in young women, as well as of vitamin B_1_ and B_12_ in some patients. Since problems such as lack of breakfast and loss of dietary diversity were also observed, it is important to educate patients about their diet.

## Figures and Tables

**Table 1:** Summary of body composition in the subjects who are 20–39 years old.

	Female (n = 1457)	Male (n = 643)	χ-Squared Test (*p*-Value)
Underweight (BMI < 18.5)	245 (16.8%)	29 (4.5%)	***p* = 9.2 × 10^−24^**
Normal weight (BMI 18.5~25)	1096 (75.2%)	482 (75%)	
Overweight (BMI 25–)	116 (8.0%)	132 (20.5%)	
Body Mass Index			
<17.5	86 (5.9%)	9 (1.4%)	
17.5–18.5	159 (10.9%)	20 (3.1%)	
18.5–25.0	1096 (75.2%)	482 (75%)	
25–30	103 (7.1%)	113 (17.6%)	
30–35	12 (0.8%)	12 (1.9%)	
35–	1 (0.07%)	7 (1.1%)	

Statistically significant difference (*p*-value < 0.05) is given in bold.

**Table 2:** The background of young women aged 20–39 years old at workplace health examinations.

	Total	Underweight (<18.5)	Normal (18.5–24.9)	Overweight (>25)	χ-Squared Test
	n = 1457	n = 245	n = 1096	n = 116	
Age (years)	28.25 ± 4.90	27.28 ± 4.46	28.26 ± 4.93 *	30.24 ± 4.90 ***	
BMI	20.74 ± 2.73	17.60 ± 0.69	20.74 ± 1.54 ***	27.36±2.26 ***	
Handgrip strength (kg)	24.10 ± 5.79	22.82 ± 5.55	24.21 ± 5.78 **	25.73 ± 5.81 ***	
sBP (mmHg)	111.5 ± 9.93	109.8 ± 9.95	111.2 ± 9.7	117.3 ± 9.9 ***	
dBP (mmHg)	68.9 ± 9.0	68.52 ± 9.18	68.7 ± 8.93	71.9 ± 8.7 **	
HbA1c (%)	5.42 ± 0.25	5.41±0.23	5.41±0.22	5.53±0.44**	
HbA1c ≧ 5.6 (n (%))	398 (27.3%)	60 (24.5%)	291 (26.6%)	47 (40.5%)	***p* = 0.0032**
HbA1c ≧ 6.5 (n (%))	2 (0.1%)	2 (0.9%)	7 (0.6%)	5 (4.3%)	***p* = 0.00057**
T-Chol (mg/dL)	183.9 ± 28.9	177.8 ± 25.2	184.1 ± 29.2 *	194.7 ± 31.2 *	
T-Chol (<180 mg/dL)	687 (47.2%)	140 (57.1%)	508 (46.3%)	39 (33.6%)	***p* = 0.000057**
Lymphocyte (/μL)	1978 ± 548	1883 ± 503	1981 ± 524 *	2148 ± 765 ***	
Lymphocyte (<1600/μL)	355 (24.3%)	83 (34%)	249 (22.7%)	23 (19.8%)	***p* = 0.00057**

Statistically significant difference (*p*-value < 0.05) is given in bold (chi-squared test) or asterisk (one-way ANOVA for parametric numerical data). * *p* < 0.05 vs. underweight, ** *p* < 0.01 vs. underweight, *** *p* < 0.001 vs. underweight. The area that is “shade” indicates the percentage of people who are larger or smaller than a certain value.

**Table 3:** The background of young women aged 20–39 years old and above 40 years old at the second checkup.

	Total	20–39 y.o.a.	>40 y.o.a.	*p*-Value
	(n = 56)	(n = 44)	(n = 12)	
Age (years)	32.41 ± 10.63	27.34 ± 3.98	51.00 ± 5.42	***p*** **= 1.60 × 10^−9^**
Body mass index (BMI)	17.02 ± 0.69	16.96 ± 0.67	17.27 ± 0.71	*p* = 0.2
BMI at 20 y.o.a.	17.6 ± 1.72	17.30 ± 1.34	18.72 ± 2.37	*p* = 0.22
Handgrip strength (kg)	23.63 ± 5.44	23.08 ± 5.76	23.66 ± 3.31	*p* = 0.062
Calf circumference (cm)	31.32 ± 1.55	31.38 ± 1.69	31.10 ± 0.86	*p* = 0.44
Βody fat (%BW)	22.16 ± 3.78	22.53 ± 3.68	20.79 ± 3.86	*p* = 0.2
Skeletal muscle index	7.12 ± 0.45	7.08 ± 0.44	7.29 ± 0.43	*p* = 0.15
Hb (g/dL)	13.02 ± 1.11	12.91 ± 1.07	13.44 ± 1.15	*p* = 0.18
Hb (<12 g/dL)	8 (14%)	7 (16%)	1 (8.3%)	*p* = 0.51
HbA1c (%)	5.48 ± 0.22	5.47 ± 0.23	5.51 ± 0.17	*p* = 0.47
HbA1c (≧5.6%)	22 (39%)	16 (36%)	6 (50%)	*p* = 0.39
TSH (μIU/mL)	1.55 ± 1.25	1.50 ± 1.05	1.74 ± 1.77	*p* = 0.21
FT_4_ (ng/dL)	1.34 ± 0.34	1.33 ± 0.36	1.22 ± 0.23	*p* = 0.68
CRP (mg/dL)	0.023 ± 0.024	0.022 ± 0.023	0.023 ± 0.018	*p* = 0.96
Prealbumin (mg/dL)	23.73 ± 4.16	23.28 ± 3.60	25.33 ± 0.26	*p* = 0.26
Prealbumin (<22 mg/dL)	18 (32%)	15 (34%)	3 (25%)	*p* = 0.55
Alb (g/dL)	4.47 ± 0.30	4.49 ± 0.31	4.38 ± 0.26	*p* = 0.25
Alb (<4 mg/dL)	3 (5.3%)	2 (4.5%)	1 (8.3%)	*p* = 0.61
Cholesterol (mg/dL)	180.93 ± 45.14	173.79 ± 21.45	207.08 ± 83.38	*p* = 0.22
Cholesterol (<180 mg/dL)	32 (57.1%)	26 (59.0%)	6 (50%)	*p* = 0.31
Lymphocyte (/μL)	1795 ± 519	1908 ± 486	1382 ± 419	***p*** **= 0.0019**
Lymphocytes (<1600/μL)	24 (43%)	14 (32%)	10 (83%)	***p*** **= 0.001**
CONUT score				
CONUT normal (0–1)	38 (68%)	33 (75%)	5 (42%)	***p*** **= 0.0017**
CONUT mild high (2–3)	18 (32%)	11 (25%)	7 (58%)	

Statistically significant difference (*p*-value < 0.05) is given in bold (chi-squared test) or normal (one-way ANOVA for parametric numerical data). The area that is “shade” indicates the percentage of people who are larger or smaller than a certain value. The CONUT score is a screening tool for detecting inadequate nutrition based on low albumin, cholesterol, and lymphocytes.

**Table 4:** The background of young women aged 20-39 years old and above 40 years old admitted to the outpatient nutritional evaluation.

	Total (n = 56)	20–39 y.o.a. (n = 44)	>40 y.o.a. (n = 12)	*p*-Value
Breakfast Skipping	16 (29%)	14 (32%)	2 (17%)	0.3
DDS low (0–3)	28 (50%)	22 (50%)	6 (50%)	0.16
DDS middle (4–6)	22 (39%)	19 (43%)	3 (25%)	
DDS high (7–10)	6 (11%)	3 (7%)	3 (25%)	
TEI (kcal)	1631 ± 431	1632 ± 399	1627 ± 536	0.97
TEI (<2050 kcal)	49 (88%)	40 (91%)	9 (75%)	0.14
TEE (kcal)	1659 ± 118	1690 ± 104	1544 ± 93	**0.00025**
TEE to TEI ratio	0.99 ± 0.28	0.97 ± 0.24	1.06 ± 0.37	0.44
Protein (g)	58.2 ± 17.4	57.4 ± 17.2	61.4 ± 17.6	0.5
Protein (<50 g)	16 (29%)	14 (32%)	2 (17%)	**5.43 × 10^−41^**
Fat (g)	56.5 ± 17.0	56.5 ± 16.7	56.5–18.1	0.99
Fat (g) (<46 g)	12 (21%)	9 (20%)	3 (25%)	0.73
Carbohydrate (g)	212 ± 57	214 ± 51	205 ± 76	0.72
Carbohydrate (<256 g)	46 (82%)	39 (89%)	7 (58%)	**0.015**
Dietary fiber (g)	10.8 ± 3.9	10.3 ± 3.4	12.5 ± 5.1	0.2
Dietary fiber (<18.9 g)	54 (96%)	42 (95%)	10 (83%)	0.15
Cholesterol (g)	277.7 ± 95.9	275.09 ± 99.8	287.5 ± 79.3	0.66
SFA (g)	17.3 ± 5.4	17.5 ± 5.3	16.9 ± 5.6	0.75
MUFA (g)	20.3 ± 6.2	20.2 ± 6.1	20.5 ± 6.6	0.91
PUFA (g)	12.3 ± 4.1	12.2 ± 3.9	12.7 ± 4.7	0.72
*n*-3 PUFA (g)	2.04 ± 0.81	2.00±0.81	2.21±0.81	0.43
*n*-3 PUFA (<1.6 g/day)	18 (32%)	15 (34%)	3 (25%)	0.55
n-6 PUFA (g)	10.23 ± 3.31	10.16 ± 3.13	10.47 ± 3.90	0.8
*n*-6 PUFA (<8 g/day)	16 (29%)	13 (30%)	3 (25%)	0.75
Fe (g)	6.11 ± 2.11	5.90 ± 1.99	6.89 ± 2.34	0.21
Fe (<10.5 g)	54 (96%)	43 (97%)	11 (92%)	0.32
Calcium (mg)	382.3 ± 143.8	385.7 ± 149.0	369.8 ± 116.3	0.71
Calcium (<650 mg)	54 (96%)	42 (95%)	12 (100%)	0.45

Statistically significant difference (*p*-value < 0.05) is given in bold (chi-squared test) or normal (one-way ANOVA for parametric numerical data). The area that is “shade” indicates the percentage of people who are larger or smaller than a certain value. Abbreviations: dietary diversity score, DDS; TEI, total energy intake; TEE, total energy expenditure; saturated fatty acids, SFAs; monounsaturated fatty acids, MUFAs; polyunsaturated fatty acids, PUFAs.

**Table 5:** The background of young women aged 20–39 years old and above 40 years old at the second checkup.

	Total	20–39 y.o.a.	>40 y.o.a.	*p*-Value
	n = 56	n = 44	n = 12	
Vitamin B_1_ intake (g)	0.90 ± 0.28	0.89 ± 0.28	0.92 ± 0.25	0.72
Vitamin B_1_ intake (<1.1 mg)	48 (86%)	38 (86%)	10 (83%)	0.79
Plasma Vitamin B_1_ (ng/mL)	31.18 ± 12.24	32.11 ± 13.33	27.75 ± 5.75	0.11
Plasma B_1_ deficiency (<24 ng/mL)	5 (8.9%)	2 (4.6%)	3 (25%)	**0.027**
Vitamin B_12_ intake (μg)	4.60 ± 2.93	4.22 ± 2.92	5.96 ± 2.53	0.064
Vitamin B_12_ intake (<2.0 μg)	10 (18%)	9 (20%)	1 (8%)	0.33
Plasma Vitamin B_12_ (pg/mL)	311.16 ± 185.25	293.20 ± 154.25	377 ± 259.60	0.32
Plasma B_12_ deficiency (<200 pg/mL)	14 (25%)	11 (25%)	3 (25%)	0.31
Folate intake (μg)	217 ± 89	204 ± 78	268 ± 106	0.08
Folate intake (<240μg)	39 (70%)	35 (80%)	4 (33%)	**0.002**
Plasma Folate (ng/mL)	8.51 ± 5.07	7.91 ± 5.05	10.73 ± 4.54	0.089
Plasma Folate deficiency (<4 ng/mL)	6 (11%)	6 (14%)	0 (0%)	0.18
Vitamin D intake (μg)	4.46 ± 3.11	4.12 ± 3.14	5.72 ± 2.65	0.1
Vitamin D intake (<8.5 μg)	52 (93%)	42 (95%)	10 (83%)	0.15
Plasma 25-OH Vitamin D (ng/mL)	11.07 ± 4.86	10.78 ± 4.18	12.13 ± 6.71	0.53
Plasma 25-OH Vitamin D deficiency (<20 ng/mL)	53 (95%)	43 (98%)	10 (83%)	**0.049**

Statistically significant difference (*p*-value < 0.05) is given in bold (chi-squared test) or normal (one-way ANOVA for parametric numerical data). The area that is “shade” indicates the percentage of people who are larger or smaller than a certain value.

## Data Availability

The datasets used and/or analyzed during the current study are available from the corresponding author upon reasonable request.
